# Fatty Acid Oxidation Disorders Detected by Newborn Screening: Clinical, Biochemical and Genetic Spectrum in a Single‐Center Cohort

**DOI:** 10.1002/jmd2.70132

**Published:** 2026-10-14

**Authors:** Elena Martín‐Hernández, Delia Barrio‐Carreras, María Martín‐Cazaña, Luis Miguel García‐Cárdaba, Carmen Martín‐Fernández, Patricia Pérez‐Mohand, Teresa Hernández Chamorro, Berta Zamora Crespo, Pedro Ruiz‐Sala, Patricia Alcaide Alonso, Pilar Quijada‐Fraile

**Affiliations:** ^1^ Departamento de Pediatría, Hospital Universitario 12 de Octubre, National Reference Center (CSUR) for Inherited Metabolic Diseases Unidad de Enfermedades Metabólicas‐Mitocondriales Madrid Spain; ^2^ Instituto de Investigación Sanitaria Hospital 12 de Octubre (imas12) Madrid Spain; ^3^ Centro de Investigación Biomédica en Red de Enfermedades Raras (CIBERER), U723, Instituto de Salud Carlos III (ISCIII) Madrid Spain; ^4^ Centro de Diagnóstico de Enfermedades Moleculares (CEDEM), Universidad Autónoma de Madrid. CIBERER, IdiPAZ Madrid Spain

**Keywords:** fatty acid oxidation disorders (FAOD), long‐chain FAOD (LC‐FAOD), medium‐chain acyl‐CoA dehydrogenase deficiency (MCADD), metabolic decompensation, neuropsychological disorders, newborn screening (NBS)

## Abstract

Newborn screening (NBS) has significantly improved early detection and survival in fatty acid oxidation disorders (FAODs). However, the expanding phenotypic spectrum challenges prognostic stratification, and long‐term morbidity remains incompletely characterized. We conducted an observational, retrospective, single‐center study of patients with FAODs identified through NBS and followed between 2011 and 2024. Clinical, biochemical, genetic, and nutritional data were extracted from medical records. Correlations between biochemical markers, enzymatic activity, and clinical outcomes were analyzed. The study was approved by the Research Committee of the Instituto de Investigación, Hospital 12 de Octubre (Madrid). Sixty‐five patients were included: 42 MCADD, 11 VLCADD, 5 LCHADD, 2 CPT1D, 3 CPT2D, 1 CACTD, and 1 MADD. Median age at last follow‐up was 7.2 years (range 0.4–13.6). Two patients died during follow‐up (overall mortality 3.1%). In MCADD, confirmatory C8 concentrations correlated with residual enzyme activity and metabolic decompensations. In VLCADD, NBS and confirmatory C14:1 levels correlated with enzyme activity but not with clinical severity. VLCADD showed marked clinical heterogeneity, whereas LCHADD patients had the highest disease burden and required the most intensive nutritional management. Overall, 40% of patients experienced at least one metabolic decompensation, occurring significantly more frequently in LC‐FAOD than in MCADD. Neuropsychological manifestations were observed in 16.9% of patients. In summary, NBS markedly improves survival in FAODs, but morbidity remains substantial, particularly in LC‐FAOD. Current biochemical markers are useful for early risk stratification but remain insufficient to predict long‐term clinical severity. These findings support individualized multidisciplinary follow‐up, including specialized nutritional care and systematic neuropsychological assessment.

## Key Points

- FAODs identified through newborn screening display a broad clinical spectrum, with substantial morbidity persisting particularly in long‐chain FAODs.
- Current biochemical markers may contribute to early risk stratification but remain insufficient to reliably predict long‐term clinical severity.
- Neuropsychological manifestations were a relevant finding in our cohort, highlighting the importance of neuropsychological assessment during long‐term follow‐up.

## 1 Introduction

Mitochondrial fatty acid oxidation disorders (FAODs) are a heterogeneous group of autosomal recessive inborn errors of metabolism affecting fatty acid transport or mitochondrial β‐oxidation. Defective fatty acid utilization particularly compromises organs with high energy demand, including liver, skeletal muscle, and heart. During catabolic stress, impaired β‐oxidation may lead to energy deficiency and accumulation of toxic intermediates, causing hypoketotic hypoglycemia, cardiomyopathy, rhabdomyolysis, hepatopathy, and sudden death [1, 2]. Diagnosis is usually based on characteristic acylcarnitine profiles and molecular confirmation [3].

Medium‐chain acyl‐CoA dehydrogenase deficiency (MCADD, OMIM #201450) is the most common FAOD in Western countries, followed by very‐long‐chain acyl‐CoA dehydrogenase deficiency (VLCADD, OMIM #201475), whereas long‐chain hydroxyacyl‐CoA dehydrogenase deficiency (LCHADD, OMIM #609016), mitochondrial trifunctional protein deficiency (MTPD, OMIM #609015), carnitine palmitoyltransferase deficiencies (CPT1D OMIM #255120; CPT2D OMIM #255110; #600649; #608836), carnitine‐acylcarnitine translocase deficiency (CACTD OMIM #212138), and multiple acyl‐CoA dehydrogenase deficiency (MADD, OMIM #231680) are rarer [3, 4, 5, 6, 7, 8].

The incorporation of tandem mass spectrometry into newborn screening (NBS) programs has markedly changed the diagnostic landscape of FAODs by enabling presymptomatic detection and early treatment [9, 10, 11, 12]. Characteristic acylcarnitine markers allow detection of the most clinically relevant disorders, including as principal markers C8 for MCADD, C14:1 for VLCADD, long‐chain hydroxyacylcarnitines for LCHADD/MTPD, elevated free carnitine and diagnostic ratios for CPT1D, and increases in C16 and C18:1 for CPT2D or CACTD [13, 14, 15, 16, 17, 18].

NBS has substantially reduced mortality and severe metabolic crises, particularly in MCADD [12, 19]. However, screening has also broadened the recognized phenotypic spectrum, especially in VLCADD, where asymptomatic or mild forms are increasingly identified [4, 7]. In contrast, other long‐chain FAODs (LC‐FAODs) such as LCHADD, MTPD, or CACTD may still be associated with significant morbidity [20, 21, 22].

Nutritional management includes avoidance of fasting and emergency regimens during illness, with dietary fat modification and medium‐chain triglyceride (MCT) supplementation in LC‐FAODs [23, 24]. New therapeutic approaches such as triheptanoin may further improve outcomes in selected patients with LC‐FAODs [25].

Despite advances, long‐term morbidity and prognostic stratification remain challenging, highlighting the need for better prognostic markers.

## 2 Objective

To describe the epidemiological, biochemical, genetic, and clinical spectrum of patients with mitochondrial FAODs detected by newborn screening (NBS) and followed at a reference center for inherited metabolic diseases.

## 3 Methods

This retrospective, observational, single‐center study included all patients with genetically confirmed mitochondrial FAODs identified through NBS between June 2011 and December 2024, who were followed at the Pediatric Unit for Inherited Metabolic Diseases, Hospital Universitario 12 de Octubre (Madrid, Spain). Newborns with abnormal screening results in whom FAOD was not confirmed were excluded.

Data extracted from electronic medical records included demographic and clinical characteristics, biochemical and genetic findings, enzymatic activity, dietary management, anthropometry, neurodevelopmental outcomes, hospital admissions, metabolic decompensations, complications, and mortality.

NBS was performed within the Regional Newborn Screening Program of Madrid. Dried blood spot samples, collected approximately 48 h after birth, were sent to the regional screening laboratory in Hospital Gregorio Marañón, where they were analyzed by non‐derivatized tandem mass spectrometry using NeoBase or NeoBase 2 kits (PerkinElmer) according to the study period. The tandem mass spectrometer used was an Acquity TQD UPLC/MS system (Waters, Milford, MA, USA). First‐tier markers, combined markers, and ratios used for FAOD screening included: C0, C2, C6, C8, C10, C10:1, C12, C12:1, C14, C14:1, C14:2, C14OH, C16, C16OH, C16:1OH, C18, C18:1, C18:2, C18OH, C18:1OH, C0/(C16 + C18), (C0 + C2 + C3 + C16 + C18 + C18:1)/citrulline, C14:1/C12, C14:1/C16, C14:1/C2, C16 + C18:1, C16OH/C16, C18OH/C18, C8/C10, and C8/C2. Cut‐off values were periodically reviewed. Abnormal screening results were reanalyzed and, when appropriate, a second dried blood spot sample was requested. Newborns with a positive NBS result were referred, according to demographic criteria, to one of four designated hospitals within the Madrid regional NBS program, including Hospital Universitario 12 de Octubre, which is also a National Reference Center for inherited metabolic diseases. Additional information on the [26] is available at the official website (https://www.comunidad.madrid/salud/cribado‐neonatal).

Confirmatory plasma samples were obtained as soon as a positive NBS result became available, at a median age of 7 days (range 3–33). In newborns who developed symptoms before the NBS result was available, biochemical investigations were initiated at the time of clinical presentation.

Confirmatory biochemical testing, including plasma acylcarnitine analysis, was performed at the Centro de Diagnóstico de Enfermedades Moleculares, Universidad Autónoma de Madrid (CEDEM). Enzyme activity in lymphocytes was determined in all patients with VLCADD and in selected patients with MCADD at the same center according to the previously described protocols [27, 28]. Molecular studies were performed at the Department of Genetics, Hospital Universitario 12 de Octubre. Diagnostic approaches evolved from Sanger sequencing to targeted next‐generation sequencing panels during the study period. Candidate genes were selected according to biochemical phenotype and HPO terms. Variants were interpreted according to ACMG/AMP guidelines using ClinVar, LOVD, HGMD Professional, population databases and in silico tools [29]. Copy‐number variants (CNVs) were assessed in NGS‐based studies using ExomeDepth (v1.1.10).

### 3.1 Follow‐Up

Symptomatic patients were evaluated as clinically indicated, whereas asymptomatic patients were routinely followed every 3 months during the first year of life, every 4–6 months until 6 years of age, and every 6–12 months thereafter. At each visit, patients were jointly reviewed by a metabolic pediatrician and a specialist dietitian, including clinical evaluation, anthropometry, and dietary assessment.

Anthropometric data were converted to *z*‐scores using the Spanish Growth Studies 2010 reference charts [30] and Fenton [31] Growth Charts www.ucalgary.ca/fenton.

Routine laboratory monitoring was performed every 6–12 months. Plasma acylcarnitine profiles, vitamins, PUFAs, and liver ultrasound were generally obtained annually. In patients with neurodevelopmental or behavioral abnormalities suggestive of an additional etiology, genetic investigations were expanded.

Patients with LC‐FAOD underwent baseline cardiological evaluation, including echocardiography, with subsequent assessments performed as clinically indicated. Patients with LCHADD underwent regular ophthalmological and neurophysiological evaluations from 6 years of age, or earlier if clinically indicated.

Formal neuropsychological assessment was not performed systematically as part of a prospective research protocol. Patients who developed neurodevelopmental, cognitive, learning, or behavioral concerns during follow‐up were referred to the neuropsychology clinic, where qualitative and quantitative assessments were performed using validated pediatric neuropsychological tests. Autism spectrum disorder and attention‐deficit/hyperactivity disorder were diagnosed by the corresponding specialists according to DSM‐5 criteria. The median age at first neuropsychological assessment was 2.06 years (IQR 1.19–2.95).

Nutritional management initially followed international recommendations and was subsequently individualized according to clinical severity and evolving evidence.

L‐carnitine supplementation was considered in selected patients with hypocarnitinemia, defined as free carnitine concentrations < 6 μmol/L during the first month of life or < 21 μmol/L thereafter.

Metabolic decompensation was defined as hospital admission lasting more than 24 h associated with compatible clinical or biochemical findings. For statistical analyses, both the absolute number of metabolic decompensations and the number of metabolic decompensations per patient‐year of follow‐up were recorded. Because these variables were highly correlated, metabolic decompensations per patient‐year were used in the correlation analyses. Neonatal decompensations occurring before newborn screening results were available were analyzed separately as symptoms before diagnosis and were not considered metabolic decompensations. Likewise, hospital admissions due to intercurrent illnesses without clear evidence of metabolic deterioration were not included in the decompensation analyses. These admissions were managed according to the patient's clinical status, with particular attention to maintaining adequate caloric intake and preventing catabolism. When oral intake was preserved, the usual diet was maintained and supplemented with carbohydrate sources in MCADD and with carbohydrate sources and MCT in LC‐FAOD. In patients with vomiting, diarrhea, or poor oral tolerance, intravenous glucose with electrolytes was administered according to age and clinical requirements, together with treatment of the underlying intercurrent illness.

Statistical analyses were performed using Stata version 16. Continuous variables were expressed as mean ± SD or median (IQR), and categorical variables as frequencies and percentages. Group comparisons and correlation analyses were performed using appropriate parametric or nonparametric tests. A two‐tailed *p* value < 0.05 was considered statistically significant.

The study was approved by the Research Committee of the Instituto de Investigación (TP24/0352), Hospital Universitario 12 de Octubre.

## 4 Results

Between 2011 and 2024, 862 144 newborns were screened in the Community of Madrid. The incidences of FAODs detected by the Regional Newborn Screening Program were 1:14369 for MCADD, 1:50714 for VLCADD, 1:123163 for LCHADD, 1:862144 for MADD, 1:57476 for primary carnitine deficiency, 1:431072 for both CPT1 and CPT2 deficiencies and 1:862144 newborns for CACTD deficiency. The overall incidence of FAODs detected by NBS was 1:8210 newborns. These patients were referred to one of the four designated hospitals within the Madrid regional NBS program for confirmatory diagnosis, treatment, and follow‐up. The present study includes patients with FAOD managed at Hospital Universitario 12 de Octubre, including some referred from other centres. Patients with primary carnitine deficiency were not included.

### 4.1 Cohort Characteristics

The study included 65 patients (52.3% male), with a median age at last follow‐up of 7.2 years (range 0.4–13.6). Diagnoses were MCADD (*n* = 42), VLCADD (*n* = 11), LCHADD (*n* = 5), CPT1D (*n* = 2), CPT2D (*n* = 3), CACTD (*n* = 1) and MADD (*n* = 1). Overall, 22 patients had LC‐FAOD, 42 MCADD and one MADD (Table 1).

**TABLE 1:** Clinical characteristics of the study cohort.

Diseases	*N* (%)	Sex (M/F)	Consanguinity *N* (%)	Prematurity *N* (%)	Day at results of NBS^a^	Age at last visit (years)^a^	Mortality *N* (%)
					Median (range)	Median (range)	
MCADD	42 (64.6)	20/22	6 (14.3)	1 (2.4)	8 (3–33)	7.6 (0.7–12.7)	0
VLCADD	11 (16.9)	7/4	2 (18.2)	2 (18.2)	5.5 (4–14)	6.7 (1.3–13.6)	0
LCHADD	5 (7.7)	4/1	1 (20)	4 (80)	7 (4–8)	3 (0.5–9)	1 (20)
CPT1D	2 (3.1)	1/1	1 (50)	1 (50)	10	8.3 (4.4–12.2)	0
CPT2D	3 (4.6)	0/3	0 (0)	0 (0)	7 (5–18)	9.7 (0.4–11.5)	0
CACTD	1 (1.5)	1/0	1 (100)	0 (0)	4	1.2	1 (100)
MADD	1 (1.5)	1/0	0 (0)	0 (0)	8	7	0
Total	65	34/31	11 (16.9)	8 (12.3)	7 (3–33)	7.2 (0.4–13.6)	2 (3.1)

Abbreviations: CACTD: carnitine‐acylcarnitine translocase deficiency; CPT1D: carnitine palmitoyltransferase 1 deficiency; CPT2D: carnitine palmitoyltransferase 2 deficiency; LCHADD: long‐chain 3‐hydroxyacyl‐CoA dehydrogenase deficiency; MADD: multiple acyl‐CoA dehydrogenase deficiency; MCADD: medium‐chain acyl‐CoA dehydrogenase deficiency; VLCADD: very‐long‐chain acyl‐CoA dehydrogenase deficiency.

^a^ Data are presented as median (range).

Consanguinity was reported in 16.9% of families. Prematurity was significantly more frequent in LC‐FAOD than in MCADD (31.8% vs. 2.4%; *p* < 0.001), particularly in LCHADD. Fourteen patients (21.5%) developed symptoms before NBS results were available, with symptom onset occurring within the first days of life in all cases. Most of these newborns had significant perinatal comorbidities, including prematurity, maternal HELLP syndrome, low birth weight, respiratory distress syndrome, or other complications, making it difficult to determine whether the initial manifestations were attributable to the underlying FAOD or to concurrent perinatal conditions. After detailed clinical review, early symptoms were considered likely attributable to the underlying FAOD in five patients (7.7%): one patient with LCHADD who presented with severe neonatal metabolic decompensation and cardiomyopathy, one with MADD, and one with MCADD who developed hypoglycemia requiring intravenous glucose, one with CPT2D who presented with severe hyperammonemia and seizures, and one with CACTD who developed hypoketotic hypoglycemia with lactic acidosis and hyperammonemia. In the remaining patients, symptoms were considered either unlikely or only possibly related to the underlying FAOD (Supplementary Table 1).

### 4.2 Biochemical Findings

In MCADD, the median C8 concentration was 3.48 μmol/L at NBS (IQR 0.72–9.23; range 0.18–26.00; reference < 0.13 μmol/L) and 2.65 μmol/L at confirmatory testing (IQR 0.59–4.48; range 0.21–15.19; reference < 0.25 μmol/L). Additional acylcarnitine species (C6, C10, C10:1) and diagnostic ratios (C8/C10 and C8/C2) at NBS and confirmatory testing are summarized in Supplementary Tables 2 and 3.

Disease‐specific acylcarnitines and diagnostic ratios were significantly correlated with C8 concentrations at NBS and confirmatory testing. Residual MCAD enzyme activity showed significant inverse correlations with all major biochemical markers, especially C6 and C8 concentrations at NBS and confirmatory testing. Several confirmatory‐study acylcarnitines were significantly associated with metabolic decompensations per patient‐year. For clarity, Figure 1 illustrates only the correlations involving C8 concentrations, residual enzyme activity, and metabolic decompensations, whereas all correlation analyses are summarized in Supplementary Table 4.

**FIGURE 1:**
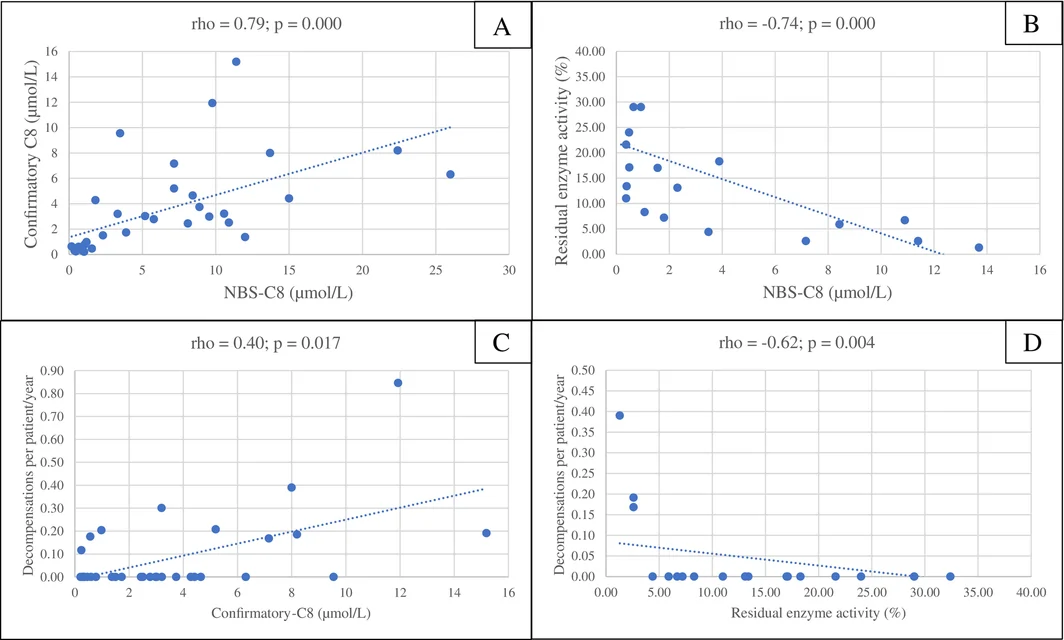
Correlations between C8 concentrations, residual MCAD enzyme activity and metabolic decompensations in MCADD.

In VLCADD, the median C14:1 concentration was 2.20 μmol/L at NBS (IQR 1.26–3.17; range 0.78–7.55; reference < 0.37 μmol/L) and 1.44 μmol/L at confirmatory testing (IQR 0.81–1.72; range 0.24–5.00; reference < 0.17 μmol/L). Additional disease‐specific acylcarnitine species (C14, C14:2, C16, C16:1, C18, C18:1 and C18:2) and the C14:1/C12:1 ratio are summarized in Supplementary Table 5. Although additional biomarkers were analyzed, C14:1 remained the principal marker and was therefore used for the main analyses.

C14:1 at NBS correlated positively with other disease‐specific acylcarnitines (C14, C16:1, C18:1) and with C14:1 at confirmatory study, and inversely with residual VLCAD enzyme activity. In contrast to MCADD, neither newborn screening nor confirmatory C14:1 concentrations, nor residual enzyme activity were significantly associated with metabolic decompensations per patient‐year (Figure 2).

**FIGURE 2:**
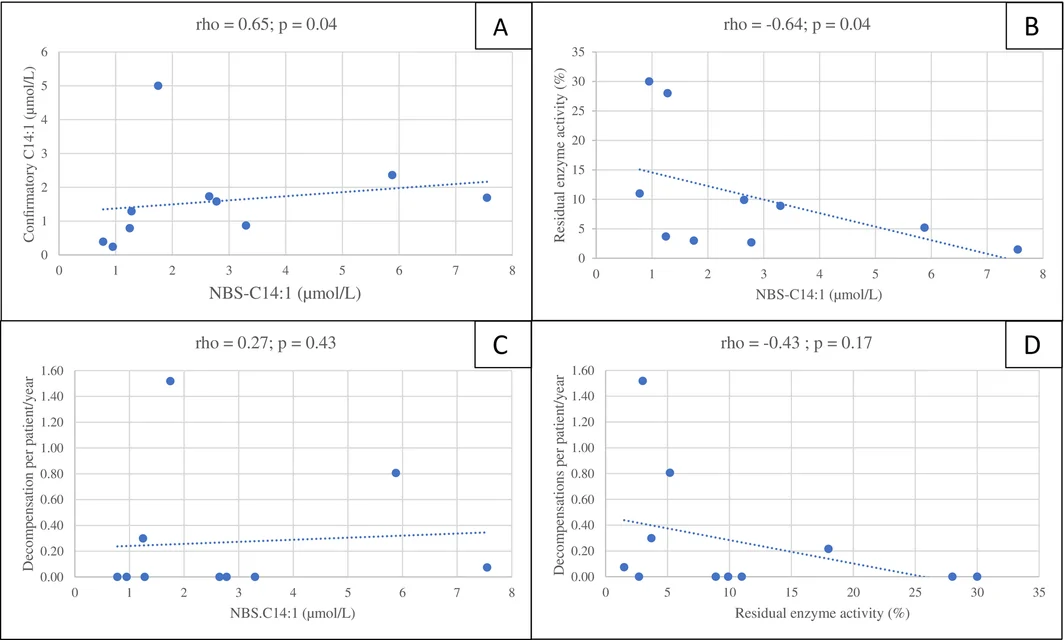
Correlations between C14:1 concentrations, residual VLCAD enzyme activity and metabolic decompensations in VLCADD.

In LCHADD, the median C16‐OH concentration decreased from 0.94 μmol/L at newborn screening (range 0.40–1.53; reference < 0.06 μmol/L) to 0.37 μmol/L at confirmatory testing (range 0.08–1.75; reference < 0.08 μmol/L). Additional disease‐specific acylcarnitine species and diagnostic ratios for LCHADD, CPT1D, CPT2D, and CACTD are summarized in Supplementary Table 6. Owing to the limited number of patients, no further correlation analyses were performed for these disorders.

### 4.3 Genetic Findings

In MCADD, 17 different *ACADM* variants were identified. The c.985A>G (p.Lys329Glu) variant was the most frequent, present in 80.9% of patients. Homozygous carriers showed significantly higher C8 concentrations, lower residual enzyme activity, and higher admission rates than patients carrying other genotypes, whereas no significant association with metabolic decompensations was observed (Table 2).

**TABLE 2:** Genetics in MCADD.

	Heterozygous^a^ (*N* = 15)	p^1^	Homozygous^a^ (*N* = 19)	p^2^	Other variants^a^ (*N* = 8)
C8‐NBS (μmol/L)	1 (0.4–3.7)	< 0.001	9.2 (7.2–12)	< 0.001	0.5 (0.4–1.0)
C8‐Confirmatory (μmol/L)	1.2 (0.4–2.6)	0.007	3.5 (3–6.3)	0.001	0.3 (0.2–0.6)
Enzyme activity (%)	15.2 (8.8–19.9)	0.011	2.6 (1.9–4.2)	0.020	28.2 (18.5–46.2)
Admissions	1.0 (0.0–4.0)	0.280	1.0 (0.0–4.0)	0.047	0.0 (0.0–2.0)
Admissions patient‐year	0.1 (0.0–0.6)	0.520	0.3 (0.0–1.4)	0.039	0.0 (0.0–0.2)
Decompensations	0.0 (0.0–2.0)	0.260	0.0 (0.0–3.0)	0.300	0.0 (0.0–1.0)
Decompensations patient‐year	0.0 (0.0–0.2)	0.240	0.0 (0.0–1.0)	0.280	0.0 (0.0–0.1)

*Note:* Genotype–Phenotype correlation.

^a^ Patients carrying the c.985A>G (p.Lys329Glu) variant. Data are presented as median (range). Kruskal Wallis test: p^1^: Difference between the homozygous and heterozygous groups; p^2^: Difference between homozygotes and patients with other variants. There were no significant differences between heterozygotes and patients with other variants in any of the variables.

Two patients presented additional genetic findings unrelated to MCADD: one carried a pathogenic NF1 variant (c.1185 + 1G>A), and another had a 15q13.2q13.3 duplication associated with neurodevelopmental features.

In VLCADD, the c.848 T>C (p.Val283Ala) variant was the most frequent *ACADVL* variant (Table 3). Homozygous patients carrying this variant remained asymptomatic despite low residual enzyme activity, whereas greater clinical heterogeneity was observed among patients with other *ACADVL* genotypes.

**TABLE 3:** Clinical, biochemical and genetic characteristics of patients with VLCADD.

Case	Sex	Age (years)	C14:1 NBS (μmol/L)	C14:1 Confirmatory (μmol/L)	Residual enzyme activity (%)	Variants in *ACADVL*	Clinical manifestations		
							Admissions	Decomp.	Clinical features
49	M	6.74	2.78	1.58	2.70	c.685C>T (p.Arg229Term) c.848 T>C (p.Val283Ala)	0	0	Asymptomatic
50	F	13.39	1.25	0.79	3.70	c.520G>A (p.Val174Met) c.1844G>A (p.Arg615Gln)	9	4	Persistent hyperCKemia
51	M	13.61	7.55	1.69	1.50	c.761G>A (p.Gly254Asp) c.761G>A (p.Gly254Asp)	1	1	Hepatic steatosis Congenital vertical talus
52	M	12.44	2.65	1.73	9.90	c.685C>T (p.Arg229Term) c.848 T>C (p.Val283Ala)	2	0	Asymptomatic
53	M	11.04	3.30	0.87	8.90	c.848 T>C (p.Val283Ala) c.848 T>C (p.Val283Ala)	1	0	Asymptomatic
54	M	9.10	0.78	0.39	11.00	c.848 T>C (p.Val283Ala) c.848 T>C (p.Val283Ala)	1	0	Asymptomatic
55	F	6.59	1.75	5.00	3.00	c.1367G>A (p.Arg456His) c.1678 + 19_1678 + 31del13 (p.?)	13	10	Recurrent metabolic decompensations
56	F	4.64	—	—	18.00	c.829_831delGAG (p.Glu277del) c.848 T>C(p.Val283Ala)	4	1	Asymptomatic
57	M	3.72	5.88	2.36	5.20	c.853G>C (p.Glu285Gln) c.1043 T>C (p.Leu348Pro)	4	3	Psychomotor delay Autism spectrum traits
58	M	1.89	1.28	1.29	28.00	c.1059_1063del (p.Lys354CysfsTer26) c.1536 T>G (p.Ser512Arg)	2	0	Asymptomatic
59	F	1.26	0.95	0.24	30.00	c.1222C>T (p.Arg408Trp) c.550G>A (p.Ala184Thr)	4	0	Mild VSD. Psychomotor delay Microcephaly

Abbreviations: C14:1: tetradecenoylcarnitine; CPK: creatine phosphokinase; F: female; M: male; NBS: newborn screening; VSD: ventricular septal defect.

Variants and their main clinical characteristics in patients with LCHADD, CPT1D, CPT2D, and CACTD are summarized in Table 4. The most severe phenotypes were observed in the patient homozygous for the *HADHA* c.453 + 1G>A variant, who died from cardiomyopathy during infancy, and in the patient with CACTD, homozygous for the *SLC25A20* c.536A>G (p.Asp179Gly) variant, who experienced recurrent severe metabolic decompensations and died at 14 months of age. Two previous siblings had died suddenly within the first 24 h of life.

**TABLE 4:** Clinical, biochemical and genetic characteristics of patients with LCHADD, CPT1D, CPT2D and CACTD.

Case	Sex	Age (years)	Disease Gene	Variants	GA BW	Previous symptoms	Clinical manifestations		
							Admissions^a^	Decomp^a^	Symptoms
1	M	9.01	LCHADD *HADHA*	c.1528G>C (p.Glu510Gln) c.1915_1918delTATC	33.0 1.900	Acidosis	3	3	Gallstones. Myopathy. MDD. ADHD
2	M	4.49	LCHADD *HADHA*	c.1528G>C (p.Glu510Gln) c.1528G>C (p.Glu510Gln)	30.4 1.350	Acidosis	6	6	Myopathy. MDD. ADHD. Autism Spectrum disorder
3	M	3.00	LCHADD *HADHA*	c.1528G>C (p.Glu510Gln) c.1528G>C (p.Glu510Gln)	34.1 2.400	No	7	3	Asymptomatic
4	M	2.00	LCHADD *HADHA*	c.1528G>C (p.Glu510Gln) c.1528G>C (p.Glu510Gln)	36.1 1.590	Hypoglycemia	11	5	Cardiomyopathy. myopathy
5	F	0.54^a^	LCHADD *HADHA*	c.453 + 1G>A (p.Met106fs) c.453 + 1G>A (p.Met106fs)	37.6 2.380	Multisystemic	3	2	Myopathy. Cardiomyopathy. Death
60	F	12.18	CPT1D *CPT1A*	c.1006G>A (p.Val336Met) c.627delT (p.Phe209Leufs*13)	36.5 1.570	No	3	3	Reye's syndrome MDD. Cognitive disability
61	V	4.44	CPT1D *CPT1A*	c.1839C>A (p.Cys313Ter) c.1839C>A (p.Cys313Ter)	39.1 3.080	Acidosis	4	2	Asymptomatic
62	F	0.39	CPT2D *CPT2*	c.1465 T>C (p.Cys489Arg) c.680C>T (p.Pro227Leu)	38.0 3.190	No	1	0	Mild hypertrophy of interventricular septum
63	F	11.48	CPT2D *CPT2*	c.587C>T (p.Pro196Leu) c.587C>T (p.Pro196Leu)	40.5 3.580	No	7	6	Myopathy
64	F	9.75	CPT2D *CPT2*	c.1547 T>C (p.Phe516Ser) c.122_130del (p.Pro41_Met43del)	39.4 2.760	Multisystemic	11	9	MDD Autism spectrum disorder
65	M	1.23^a^	CACTD *SLC25A20*	c.536A>G (p.Asp179Gly) c.536A>G (p.Asp179Gly)	39.0 3.480	Multisystemic	3	3	Failure to thrive Death

Abbreviations: ADHD: attention‐deficit/hyperactivity disorder; BW: birth weight; CACTD: Carnitine‐acylcarnitine translocase deficiency; CPT1D: Carnitine palmitoyltransferase 1A deficiency; CPT2D: Carnitine palmitoyltransferase 2 deficiency; F: female; GA: gestational age; LCHADD: long‐chain 3‐hydroxyacyl‐CoA dehydrogenase deficiency; M: male; MDD: mild developmental delay; multisystemic (several of them): cardiomyopathy, hepatopathy, acidosis, hyperlactacidemia, hypoglycemia, hyperammonemia, high CK.

^a^ Age at death; *number of admissions and decompensations.

### 4.4 Nutritional Management and Growth

Patients with MCADD received breast milk or mixed feeding during infancy, followed by a healthy diet with avoidance of prolonged fasting. During follow‐up, 25 patients (59.5%) received L‐carnitine supplementation at some point because of secondary hypocarnitinemia, at a mean dose of 46.2 ± 17.7 mg/kg/day.

In LC‐FAOD, dietary treatment was tailored according to clinical severity and included MCT‐enriched formulas, prolonged nocturnal feeding in patients with LCHADD, bedtime snacks rich in complex carbohydrates in selected patients with greater metabolic instability, and particular attention to maintaining adequate protein intake, generally within the upper normal range for age. In one patient with severe LCHADD who developed necrotizing enterocolitis, skimmed breast milk was successfully used for 4 months. In one patient with severe VLCADD, cow's milk protein intolerance and sucrase‐isomaltase deficiency precluded the use of standard MCT‐enriched formulas, contributing to recurrent metabolic decompensations and ultimately requiring gastrostomy feeding and compassionate‐use triheptanoin. Compassionate‐use triheptanoin was also administered to one patient with CPT2D because of recurrent metabolic decompensations despite optimized conventional treatment. During follow‐up, selected patients with hypocarnitinemia received L‐carnitine supplementation at some point, at a mean dose of 18.4 ± 16.5 mg/kg/day.

At the last available follow‐up, patients with LCHADD had significantly shorter permitted fasting intervals than those with VLCADD (*p* = 0.04) and received a higher proportion of dietary MCT (*p* = 0.004), reflecting the greater clinical severity of this subgroup (Table 5).

**TABLE 5:** Nutritional management in patients with long‐chain fatty acid oxidation disorders.

LC‐FAOD (*N* = 22)	Age at last follow‐up (years)	Maximum fasting interval (h)	Energy (kcal/kg/d)	LCT (%)	MCT (%)	Proteins (%)	PUFAs (%)
LCHADD (*N* = 5) (x̄ ± DS)	3.8 ± 3.2	4.7 ± 1.4	77.2 ± 13.2	10.3 ± 1.9	31.4 ± 7.4	16.6 ± 3.1	3.2 ± 2.1
VLCADD (*N* = 11) (x̄ ± DS)	7.7 ± 4.5	7.6 ± 2.4	62.2 ± 26.9	11.6 ± 2.6	18.2 ± 6.9	18.2 ± 3.4	3.1 ± 2.0
CPT1D. CPT2D. CACTD (*N* = 6) (x̄ ± DS)	6.6 ± 5.2	5.4 ± 1.5	79.2 ± 25.9	10.1 ± 3.8	21.8 ± 4.9	18.4 ± 2.4	2.0 ± 0.8
Total (*N* = 22)	6.5 ± 4.6	6.4 ± 2.3	70.3 ± 24.6	10.8 ± 2.8	22.2 ± 8.2	17.7 ± 2.9	2.9 ± 1.8

*Note:* Data are presented as mean ± SD.

Abbreviations: CACTD, carnitine‐acylcarnitine translocase deficiency; CPT1D, carnitine palmitoyltransferase 1 deficiency; CPT2D, carnitine palmitoyltransferase 2 deficiency; LC‐FAOD, long‐chain fatty acid oxidation disorders; LCHADD, long‐chain 3‐hydroxyacyl‐CoA dehydrogenase deficiency; LCT, long‐chain triglycerides; MCT, medium chain triglycerides; PUFAs, polyunsaturated fatty acids; VLCADD, very‐long‐chain acyl‐CoA dehydrogenase deficiency.

Anthropometric parameters at the last available visit remained within the reference range in both the LC‐FAOD and MC‐FAOD groups (Supplementary Table 7).

### 4.5 Hospital Admissions, Metabolic Decompensations and Complications

Overall, 26 patients (40.0%) experienced at least one metabolic decompensation. Metabolic decompensations were significantly more frequent in patients with LC‐FAOD than in those with MCADD (68.2% vs. 23.8%; *p* < 0.001). The highest proportions of affected patients were observed in CACTD (100%), LCHADD (80.0%), and CPT2D (66.7%), whereas most patients with MCADD remained free of metabolic crises.

In MCADD, most patients remained asymptomatic with normal growth and school performance. No cardiomyopathy or myopathy was observed, and only two patients developed mild and transitory hepatic abnormalities. Neuropsychological disorders were identified in 14.3% of patients, including developmental and language delay, attentional difficulties, autism spectrum traits, obsessive behaviors, eating disorders, and impaired social cognition, even in patients with high cognitive abilities.

Among VLCADD patients (Table 3), one patient required highly individualized nutritional management and gastrostomy because of recurrent metabolic instability. One patient (9.1%) presented autism spectrum traits.

In LCHADD, myopathy occurred in 80% of patients and cardiomyopathy in 40%. No patient developed liver involvement, retinopathy, or neuropathy. Attention‐deficit/hyperactivity disorder was identified in two patients (Table 4).

Among carnitine‐cycle disorders (Table 4), one patient with CPT1D presented with Reye syndrome, whereas one patient with CPT2D developed autism spectrum disorder. The patient with CACTD experienced recurrent severe metabolic decompensations and died at 14 months of age.

Overall, heterogeneous neuropsychological manifestations were identified in 16.9% of the cohort, involving multiple cognitive and behavioral domains, including attention, language, learning, executive function and social cognition.

## 5 Discussion

This study provides a comprehensive characterization of the clinical, biochemical, genetic, and nutritional spectrum of patients with FAODs identified through NBS and followed at a national reference center. As reported in other European cohorts [10, 11], MCADD was the most frequent disorder, although LC‐FAODs accounted for a relatively high proportion of our cohort.

Prematurity and severe neonatal presentation were more frequent among LC‐FAOD patients, particularly in LCHADD, consistent with previous reports [21, 32]. Despite the benefits of NBS, severe early‐onset phenotypes may still manifest before screening results become available. In our cohort, three LC‐FAOD patients presented with severe multisystem disease during the neonatal period, resulting in two deaths in infancy. Mortality among LC‐FAOD patients reached 9%, whereas no deaths occurred in MCADD, yielding an overall mortality of 3.1%, comparable to that reported in other NBS cohorts [11].

The favorable impact of NBS on MCADD prognosis is well established [12, 19, 33] and was confirmed in our cohort, where no deaths occurred and most patients remained asymptomatic with normal growth and school performance. Confirmatory C8 concentrations correlated with residual enzyme activity and metabolic decompensations, supporting previous observations that biochemical severity may improve early risk stratification [27, 34]. Interestingly, confirmatory acylcarnitine concentrations correlated more closely with clinical outcomes than NBS values, suggesting that postnatal biochemical characterization may provide additional prognostic information. Although confirmatory studies are routinely performed for diagnosis, their prognostic value has received little attention and warrants evaluation in larger prospective cohorts.

Homozygosity for the common c.985A>G variant was associated with higher C8 concentrations and lower enzyme activity, consistent with other studies [8, 35]. However, genotype did not completely predict clinical outcome, reinforcing the concept that FAODs identified through NBS represent a broad phenotypic spectrum influenced by multiple genetic and environmental factors.

In VLCADD, C14:1 concentrations correlated with residual VLCAD enzyme activity but did not reliably predict clinical outcome, consistent with previous reports [4, 7, 28]. The c.848T>C (p.Val283Ala) variant was the most frequent ACADVL variant in our cohort, accounting for 31.8% of alleles. This frequency is comparable to that reported in other NBS cohorts [4, 7] and therefore does not suggest population‐specific enrichment. As previously reported, this variant, particularly in homozygosity, is generally associated with residual enzyme activity and a relatively mild clinical phenotype, although metabolic decompensations may still occur. These findings emphasize the limitations of currently available prognostic markers and the need for improved tools for early severity prediction. Recent studies have proposed additional biomarkers, including neonatal C18:2‐carnitine concentrations, which may improve discrimination between mild and severe VLCADD phenotypes [36]. Interestingly, the only patient in our cohort with elevated C18:2‐carnitine concentrations was also the one with the highest number of metabolic decompensations. However, as this observation is based on a single patient, no conclusions can be drawn regarding its prognostic significance.

LCHADD patients showed the highest rates of decompensations, myopathy, and cardiomyopathy, in agreement with previous studies demonstrating persistent long‐term morbidity despite early diagnosis and treatment [21, 23]. Neither retinopathy nor peripheral neuropathy was observed, although the relatively young age of the cohort may have limited the detection of these late‐onset complications.

As NBS has reduced mortality and severe neurological sequelae in FAODs, attention has shifted towards subtle long‐term neuropsychological outcomes. Waisbren et al. [37] described cognitive and developmental impairments in patients with these disorders, while Everard et al. demonstrated that NBS markedly reduces severe neurological sequelae but does not completely prevent milder cognitive and behavioral difficulties. In our cohort, neuropsychological disorders were identified in 16.9% of patients. Attention‐deficit disorders were observed in 6.1%, similar to the general Spanish pediatric population [38], whereas autism spectrum disorder was identified in 4.6%, exceeding the reported prevalence of approximately 1.3% among Spanish schoolchildren [39]. However, these findings should be interpreted with caution. Neuropsychological assessment was not performed systematically but only in patients who developed cognitive, learning, or behavioral concerns during follow‐up. In addition, the relatively small sample size precludes reliable comparisons with the general pediatric population. Potential alternative contributors were also identified: two patients with MCADD had concomitant genetic diagnoses associated with neurodevelopmental features and their neurodevelopmental abnormalities were not considered potentially attributable to MCADD; two patients with LCHADD who developed ADHD had significant perinatal complications, including prematurity and maternal HELLP syndrome; and the patient with CPT2D and autism spectrum disorder experienced severe neonatal metabolic decompensation attributable to the underlying FAOD before NBS results were available. Given the small number of patients and the heterogeneity of these potential contributing factors, no formal association between neuropsychological outcomes and early metabolic events or subsequent decompensations was assessed. Nevertheless, these findings highlight the need for standardized neuropsychological assessment in prospective follow‐up studies to better define the prevalence, spectrum, and determinants of neuropsychological complications in FAOD.

Nutritional management remains the cornerstone of FAOD treatment, particularly in long‐chain disorders. During the study period, recommendations evolved substantially as evidence emerged regarding fasting tolerance, dietary fat composition, protein intake, and alternative energy substrates [20, 24, 25, 32, 40, 41, 42]. Recently published international LC‐FAOD guidelines further emphasize individualized management according to disease severity, clinical course, and metabolic stability [43]. Our cohort reflects this evolving approach, particularly in patients with LCHADD, who required shorter fasting intervals and higher dietary MCT intake because of their greater metabolic instability, and in selected patients with more complex clinical courses, in whom nutritional management included strategies such as skimmed breast milk or compassionate‐use triheptanoin. Although our experience with triheptanoin is limited to two patients (one with VLCADD and one with CPT2 deficiency), both experienced a marked reduction in metabolic decompensations following treatment initiation, in line with recent reports [25, 42, 44]. While our experience is insufficient to define standardized indications, it illustrates how individualized nutritional and pharmacological management may optimize long‐term outcomes in selected patients.

Growth parameters remained within normal ranges at the last available visit, consistent with recent reports [45]. This was achieved through multidisciplinary follow‐up including regular assessment by a specialist metabolic dietitian, enabling individualized dietary adjustments according to growth and metabolic requirements.

The main limitations of this study are its retrospective design, the small number of patients in some disease groups, and the young age of many individuals, which may underestimate late‐onset complications. Its main strength is the long‐term follow‐up of a well‐characterized single‐center cohort managed by the same multidisciplinary team.

## 6 Conclusions

Newborn screening has transformed the prognosis of patients with FAOD by enabling early diagnosis and substantially reducing severe morbidity and mortality. However, marked clinical heterogeneity persists, particularly among long‐chain FAOD, highlighting the need for improved prognostic biomarkers and increasingly individualized long‐term management. Our findings illustrate how multidisciplinary follow‐up, integrating clinical, biochemical, nutritional, and neuropsychological assessment, has become essential to optimize long‐term outcomes in this evolving population. Future collaborative longitudinal studies will be crucial to refine risk stratification, validate novel prognostic biomarkers, and evaluate emerging disease‐modifying therapies.

## Author Contributions

Conception and design: Elena Martín‐Hernández, Pilar Quijada‐Fraile, Delia Barrio‐Carreras. Clinical data collection: Teresa Hernández Chamorro. Supervision of the data collection: Pilar Quijada‐Fraile, Delia Barrio‐Carreras, Elena Martín‐Hernández. Biochemical analysis and interpretation: Patricia Alcaide Alonso, Pedro Ruiz‐Sala. Neuropsychological studies: Berta Zamora Crespo. Nutritional data: Delia Barrio‐Carreras. Writing and editing: Elena Martín‐Hernández, Pilar Quijada‐Fraile. All authors contributed to the analysis and interpretation of the results, critically revised the manuscript, and approved the final version for submission.

## Funding

The authors have nothing to report.

## Ethics Statement

This study was approved by the Research Committee of the Instituto de Investigación (TP24/0352), Hospital 12 de Octubre (imas 12), Madrid. All procedures were in accordance with the Helsinki declaration revised in 2000. Individual informed consent was waived due to the observational nature of the study.

## Conflicts of Interest

The authors declare no conflicts of interest.

## Supporting information


**Supplementary Table 1.** Patients presenting with symptoms before newborn screening results were available.

**Supplementary Table 2.** Individual newborn screening, confirmatory biochemical, residual enzyme activity and clinical data in patients with MCADD.

**Supplementary Table 3.** Descriptive statistics of biochemical, enzymatic and clinical variables in patients with MCADD.

**Supplementary Table 4.** Spearman correlations between biochemical markers, residual enzyme activity, and metabolic decompensations in MCADD.

**Supplementary Table 5.** Individual newborn screening, confirmatory biochemical and residual enzyme activity data in patients with VLCADD.

**Supplementary Table 6.** Newborn screening markers in patients with LCHADD, CPT1D, CPT2D and CACTD.

**Supplementary Table 7.** Anthropometric parameters at birth and last available visit.

## Data Availability

The data that support the findings of this study are available from the corresponding author upon reasonable request due to legal and ethical reasons.

## References

[1] S. M. Houten , S. Violante , F. V. Ventura , and R. J. Wanders , “The Biochemistry and Physiology of Mitochondrial Fatty Acid β‐Oxidation and Its Genetic Disorders,” Annual Review of Physiology 78 (2016): 23–44, 10.1146/annurev-physiol-021115-105045. 26474213

[2] J. L. Merritt, 2nd , M. Norris , and S. Kanungo , “Fatty Acid Oxidation Disorders,” Annals of Translational Medicine 6, no. 24 (2018): 473, 10.21037/atm.2018.10.57. PMC6331364 30740404

[3] C. R. Ferreira , S. Rahman , M. Keller , J. Zschocke , and ICIMD Advisory Group , “An International Classification of Inherited Metabolic Disorders (ICIMD),” Journal of Inherited Metabolic Disease 44, no. 1 (2021): 164–177, 10.1002/jimd.12348. PMC9021760 33340416

[4] M. Al Bandari , L. Nagy , V. Cruz , S. Hewson , A. Hossain , and M. Inbar‐Feigenberg , “Management and Outcomes of Very Long‐Chain Acyl‐CoA Dehydrogenase Deficiency (VLCAD Deficiency): A Retrospective Chart Review,” International Journal of Neonatal Screening 10, no. 2 (2024): 29, 10.3390/ijns10020029. PMC11036265 38651394

[5] D. Marsden , C. L. Bedrosian , and J. Vockley , “Impact of Newborn Screening on the Reported Incidence and Clinical Outcomes Associated With Medium‐ and Long‐Chain Fatty Acid Oxidation Disorders,” Genetics in Medicine 23, no. 5 (2021): 816–829, 10.1038/s41436-020-01070-0. PMC8105167 33495527

[6] H. Rocha , D. Castiñeiras , C. Delgado , et al., “Birth Prevalence of Fatty Acid β‐Oxidation Disorders in Iberia,” JIMD Reports 16 (2014): 89–94, 10.1007/8904_2014_324. PMC4221301 25012579

[7] V. Rovelli , F. Manzoni , K. Viau , M. Pasquali , and N. Longo , “Clinical and Biochemical Outcome of Patients With Very Long‐Chain Acyl‐CoA Dehydrogenase Deficiency,” Molecular Genetics and Metabolism 127, no. 1 (2019): 64–73, 10.1016/j.ymgme.2019.04.001. 31031081

[8] M. Ruoppolo , C. Cereda , T. Giovanniello , et al., “Chain Acyl‐CoA Dehydrogenase Deficiency (MCADD) Newborn Screening in Italy: Five Years' Experience From a Nationwide Program,” International Journal of Neonatal Screening 11, no. 4 (2025): 86, 10.3390/ijns11040086. PMC12550940 41133698

[9] T. G. Derks , D. J. Reijngoud , H. R. Waterham , et al., “The Natural History of Medium‐Chain Acyl CoA Dehydrogenase Deficiency in The Netherlands: Clinical Presentation and Outcome,” Journal of Pediatrics 148, no. 5 (2006): 665–670, 10.1016/j.jpeds.2005.12.028. 16737882

[10] E. Everard , H. Laeremans , F. Boemer , et al., “Impact of Newborn Screening for Fatty Acid Oxidation Disorders on Neurological Outcome: A Belgian Retrospective and Multicentric Study,” European Journal of Paediatric Neurology 49 (2024): 60–65, 10.1016/j.ejpn.2024.02.003. 38377647

[11] P. Janeiro , R. Jotta , R. Ramos , et al., “Follow‐Up of Fatty Acid β‐Oxidation Disorders in Expanded Newborn Screening Era,” European Journal of Pediatrics 178, no. 3 (2019): 387–394, 10.1007/s00431-018-03315-2. 30617651

[12] B. Wilcken , M. Haas , P. Joy , et al., “Outcome of Neonatal Screening for Medium‐Chain Acyl‐CoA Dehydrogenase Deficiency in Australia: A Cohort Study,” Lancet 369, no. 9555 (2007): 37–42, 10.1016/S0140-6736(07)60029-4. 17208640

[13] I. Bernhardt , E. Glamuzina , L. K. Dowsett , et al., “Genotype‐Phenotype Correlations in CPT1A Deficiency Detected by Newborn Screening in Pacific Populations,” JIMD Reports 63, no. 4 (2022): 322–329, 10.1002/jmd2.12271. PMC9259392 35822099

[14] M. L. Couce , D. E. Castiñeiras , J. D. Moure , et al., “Relevance of Expanded Neonatal Screening of Medium‐Chain Acyl Co‐a Dehydrogenase Deficiency: Outcome of a Decade in Galicia (Spain),” JIMD Reports 1 (2011): 131–136, 10.1007/8904_2011_28. PMC3509827 23430840

[15] N. D. Leslie and S. Saenz‐Ayala , “Very Long‐Chain Acyl‐Coenzyme A Dehydrogenase Deficiency,” in GeneReviews [Internet], ed. M. P. Adam , J. Feldman , G. M. Mirzaa , R. A. Pagon , S. E. Wallace , and A. Amemiya (University of Washington, Seattle, 2009). 20301763

[16] Á. Martín‐Rivada , L. Palomino Pérez , P. Ruiz‐Sala , et al., “Diagnosis of Inborn Errors of Metabolism Within the Expanded Newborn Screening in the Madrid Region,” JIMD Reports 63, no. 2 (2022): 146–161, 10.1002/jmd2.12265. PMC8898721 35281663

[17] P. Prasun , M. K. LoPiccolo , and I. Ginevic , “Long‐Chain Hydroxyacyl‐CoA Dehydrogenase Deficiency / Trifunctional Protein Deficiency,” in GeneReviews [Internet], ed. M. P. Adam , J. Feldman , G. M. Mirzaa , R. A. Pagon , S. E. Wallace , and A. Amemiya (University of Washington, Seattle, 2022). 36063482

[18] G. Tajima , K. Hara , M. Tsumura , et al., “Newborn Screening for Carnitine Palmitoyltransferase II Deficiency Using (C16+C18:1)/C2: Evaluation of Additional Indices for Adequate Sensitivity and Lower False‐Positivity,” Molecular Genetics and Metabolism 122, no. 3 (2017): 67–75, 10.1016/j.ymgme.2017.07.011. 28801073

[19] B. Wilcken , “Fatty Acid Oxidation Disorders: Outcome and Long‐Term Prognosis,” Journal of Inherited Metabolic Disease 33, no. 5 (2010): 501–506, 10.1007/s10545-009-9001-1. 20049534

[20] A. Kritzer , S. Tarrant , K. Sussman‐Karten , and K. Barbas , “Use of Skimmed Breast Milk for an Infant With a Long‐Chain Fatty Acid Oxidation Disorder: A Novel Therapeutic Intervention,” JIMD Reports 55, no. 1 (2020): 44–50, 10.1002/jmd2.12152. PMC7463058 32905135

[21] U. Mütze , A. Ottenberger , F. Gleich , et al., “Neurological Outcome in Long‐Chain Hydroxy Fatty Acid Oxidation Disorders,” Annals of Clinical and Translational Neurology 11, no. 4 (2024): 883–898, 10.1002/acn3.52002. PMC11021608 38263760

[22] I. Vitoria , E. Martín‐Hernández , L. Peña‐Quintana , et al., “Carnitine‐Acylcarnitine Translocase Deficiency: Experience With Four Cases in Spain and Review of the Literature,” JIMD Reports 20 (2015): 11–20, 10.1007/8904_2014_382. PMC4375124 25614308

[23] U. Spiekerkoetter , M. Lindner , R. Santer , et al., “Treatment Recommendations in Long‐Chain Fatty Acid Oxidation Defects: Consensus From a Workshop,” Journal of Inherited Metabolic Disease 32, no. 4 (2009): 498–505, 10.1007/s10545-009-1126-8. 19452263

[24] S. C. Van Calcar , M. Sowa , F. Rohr , et al., “Nutrition Management Guideline for Very‐Long Chain Acyl‐CoA Dehydrogenase Deficiency (VLCAD): An Evidence‐ and Consensus‐Based Approach,” Molecular Genetics and Metabolism 131, no. 1‐2 (2020): 23–37, 10.1016/j.ymgme.2020.10.001. 33093005

[25] J. Vockley , B. K. Burton , G. Berry , et al., “Triheptanoin for the Treatment of Long‐Chain Fatty Acid Oxidation Disorders: Final Results of an Open‐Label, Long‐Term Extension Study,” Journal of Inherited Metabolic Disease 46, no. 5 (2023): 943–955, 10.1002/jimd.12640. 37276053

[26] Madrid Regional Newborn Screening Program , “Comunidad de Madrid,” accessed July 7, 2026, available, https://www.comunidad.madrid/salud/cribado‐neonatal.

[27] P. Alcaide , I. Ferrer‐López , L. Gutierrez , et al., “Lymphocyte Medium‐Chain Acyl‐CoA Dehydrogenase Activity and Its Potential as a Diagnostic Confirmation Tool in Newborn Screening Cases,” Journal of Clinical Medicine 11, no. 10 (2022): 2933, 10.3390/jcm11102933. PMC9145342 35629059

[28] B. Merinero , P. Alcaide , E. Martín‐Hernández , et al., “Four Years' Experience in the Diagnosis of Very Long‐Chain Acyl‐CoA Dehydrogenase Deficiency in Infants Detected in Three Spanish Newborn Screening Centers,” JIMD Reports 39 (2018): 63–74, 10.1007/8904_2017_40. PMC5953901 28755359

[29] S. Richards , N. Aziz , S. Bale , et al., “Standards and Guidelines for the Interpretation of Sequence Variants: A Joint Consensus Recommendation of the American College of Medical Genetics and Genomics and the Association for Molecular Pathology,” Genetics in Medicine 17, no. 5 (2015): 405–424, 10.1038/gim.2015.30. PMC4544753 25741868

[30] E. Sánchez González , A. Carrascosa Lezcano , J. M. Fernández García , A. Ferrández Longás , D. López de Lara , and J. P. López‐Siguero , “Estudios españoles de Crecimiento: Situación Actual, Utilidad y Recomendaciones de Uso [Spanish Growth Studies: The Current Situation, Their Effectiveness and Recommendations for Their Use],” Anales de Pediatría (Barcelona) 74, no. 3 (2011): 193.e1–193.e16, Spanish, 10.1016/j.anpedi.2010.10.005. 21237733

[31] T. R. Fenton , “Third Generation Preterm Growth Charts,” accessed July 20, 2025, available, http://www.ucalgary.ca/fenton.

[32] U. Spiekerkoetter , M. Lindner , R. Santer , et al., “Management and Outcome in 75 Individuals With Long‐Chain Fatty Acid Oxidation Defects: Results From a Workshop,” Journal of Inherited Metabolic Disease 32, no. 4 (2009): 488–497, 10.1007/s10545-009-1125-9. 19399638

[33] U. Mütze , J. Stengel , F. Gleich , et al., “Long‐Term Outcomes of Adolescents and Young Adults Identified by Metabolic Newborn Screening,” Pediatrics 155, no. 4 (2025): e2024068293, 10.1542/peds.2024-068293. 40101767

[34] S. Tucci , C. Wagner , S. C. Grünert , et al., “Genotype and Residual Enzyme Activity in Medium‐Chain Acyl‐CoA Dehydrogenase (MCAD) Deficiency: Are Predictions Possible?,” Journal of Inherited Metabolic Disease 44, no. 4 (2021): 916–925, 10.1002/jimd.12368. 33580884

[35] G. Gramer , G. Haege , J. Fang‐Hoffmann , et al., “Medium‐Chain Acyl‐CoA Dehydrogenase Deficiency: Evaluation of Genotype‐Phenotype Correlation in Patients Detected by Newborn Screening,” JIMD Reports 23 (2015): 101–112, 10.1007/8904_2015_439. PMC4484909 25940036

[36] M. Schwantje , R. E. Maase , E. Dekkers , et al., “Direct Prediction of VLCADD Severity Using Newborn Screening Analyte Data,” Journal of Inherited Metabolic Disease 49, no. 2 (2026): e70143, 10.1002/jimd.70143. PMC12912846 41702539

[37] S. E. Waisbren , Y. Landau , J. Wilson , and J. Vockley , “Neuropsychological Outcomes in Fatty Acid Oxidation Disorders: 85 Cases Detected by Newborn Screening,” Developmental Disabilities Research Reviews 17, no. 3 (2013): 260–268, 10.1002/ddrr.1119. PMC4137760 23798014

[38] F. Catalá‐López , S. Peiró , M. Ridao , G. Sanfélix‐Gimeno , R. Gènova‐Maleras , and M. A. Catalá , “Prevalence of Attention Deficit Hyperactivity Disorder Among Children and Adolescents in Spain: A Systematic Review and Meta‐Analysis of Epidemiological Studies,” BMC Psychiatry 12 (2012): 168, 10.1186/1471-244X-12-168. PMC3534011 23057832

[39] Ministerio de Educación , Formación Profesional y Deportes. Estadística de las Enseñanzas no Universitarias. Alumnado con Necesidad Específica de Apoyo Educativo. Curso 2024–2025 (Ministerio de Educación, Formación Profesional y Deportes, 2026), available, https://www.educacionfpydeportes.gob.es/dam/jcr:27b3a08d‐2d3d‐41fd‐90e2‐30b32e6815cc/2024‐25‐acneae‐nota‐resumen.pdf.

[40] M. B. Gillingham , G. Elizondo , A. Behrend , et al., “Higher Dietary Protein Intake Preserves Lean Body Mass, Lowers Liver Lipid Deposition, and Maintains Metabolic Control in Participants With Long‐Chain Fatty Acid Oxidation Disorders,” Journal of Inherited Metabolic Disease 42, no. 5 (2019): 857–869, 10.1002/jimd.12155. PMC7452215 31295363

[41] C. Alonso‐Diaz , D. Escudero‐Vieco , P. Quijada‐Fraile , et al., “Nutrition With Skimmed Breast Milk in an Infant With Long Chain 3‐Hydroxyacyl‐coA Dehydrogenase Deficiency,” JIMD Reports 66, no. 3 (2025): e70018, 10.1002/jmd2.70018. PMC12056594 40337100

[42] J. Vockley , B. Burton , G. Berry , et al., “Effects of Triheptanoin (UX007) in Patients With Long‐Chain Fatty Acid Oxidation Disorders: Results From an Open‐Label, Long‐Term Extension Study,” Journal of Inherited Metabolic Disease 44, no. 1 (2021): 253–263, 10.1002/jimd.12313. PMC7891391 32885845

[43] S. C. Grünert , K. Bhattacharya , D. Karall , et al., “International Guideline on the Diagnosis, Treatment, and Monitoring of Long‐Chain Fatty Acid Oxidation Disorders (LC‐FAOD),” Journal of Inherited Metabolic Disease 49, no. 5 (2026): e70251, 10.1002/jimd.70251. PMC13601914 42779363

[44] R. Forsyth and J. Vockley , “A Pharmacological Profile of Triheptanoin for the Treatment of Long‐Chain Fatty Acid Oxidation Disorders,” Expert Review of Clinical Pharmacology 18, no. 7 (2025): 449–460, 10.1080/17512433.2025.2528835. 40633017

[45] M. Wasiewicz‐Gajdzis , M. Jamka , J. Geltz , et al., “Anthropometric Parameters in Patients With Fatty Acid Oxidation Disorders: A Case‐Control Study, Systematic Review and Meta‐Analysis,” Health 10, no. 12 (2022): 2405, 10.3390/healthcare10122405. PMC9777909 36553929

