# Supplementary material for: Fatty Acid Oxidation Disorders Detected by Newborn Screening: Clinical, Biochemical and Genetic Spectrum in a Single‐Center Cohort
Source: JIMD Rep. 2026 Oct 14;67(6):e70132. doi: 10.1002/jmd2.70132 (PMC13662738; doi:10.1002/jmd2.70132)
Supplement: Supplementary file 1 [file JMD2-67-e70132-s001.docx]

Supplementary Table 3. Descriptive statistics of biochemical, enzymatic and clinical variables in patients with MCADD

|  | C6-NBS  µmol/L | C8-NBS  µmol/L | C10-NBS  µmol/L | C10:1-NBS  µmol/L | C8/C10-NBS | C8/C2-NBS | C6-CS  µmol/L | C8-CS  µmol/L | C10-CS  µmol/L | C10:1-CS  µmol/L | C8/C10-CS | EA (%) | A/P/Y | D/P/Y |
| --- | --- | --- | --- | --- | --- | --- | --- | --- | --- | --- | --- | --- | --- | --- |
| N | 28 | 39 | 31 | 24 | 32 | 21 | 28 | 36 | 13 | 21 | 12 | 20 | 42 | 42 |
| Mean | 1.04 | 5.75 | 0.67 | 0.37 | 8.58 | 0.27 | 0.98 | 3.40 | 0.51 | 0.72 | 8.04 | 16.25 | 0.24 | 0.07 |
| SD | 0.73 | 6.22 | 0.39 | 0.29 | 6.11 | 0.27 | 1.06 | 3.58 | 0.30 | 0.71 | 5.77 | 13.99 | 0.34 | 0.16 |
| Median | 0.88 | 3.48 | 0.64 | 0.30 | 10.94 | 0.25 | 0.75 | 2.65 | 0.47 | 0.48 | 9.82 | 13.25 | 0.12 | 0.00 |
| p25 | 0.44 | 0.72 | 0.35 | 0.15 | 1.60 | 0.04 | 0.24 | 0.59 | 0.38 | 0.35 | 1.85 | 6.50 | 0.00 | 0.00 |
| p75 | 1.60 | 9.23 | 0.84 | 0.41 | 14.06 | 0.35 | 1.21 | 4.48 | 0.63 | 0.71 | 11.72 | 22.20 | 0.35 | 0.00 |
| Min | 0.08 | 0.18 | 0.25 | 0.08 | 0.72 | 0.01 | 0.12 | 0.21 | 0.14 | 0.11 | 1.12 | 1.30 | 0.00 | 0.00 |
| Max | 2.46 | 26.00 | 2.01 | 1.27 | 17.36 | 0.80 | 4.95 | 15.19 | 1.25 | 2.65 | 16.56 | 60.00 | 1.40 | 0.85 |

NBS: Newborn screening; CS: Confirmatory study; EA: Residual enzyme activity; A/P/Y: Admissions per patient/year; D/P/Y: Decompensations per patient/year
