# Supplementary material for: Fatty Acid Oxidation Disorders Detected by Newborn Screening: Clinical, Biochemical and Genetic Spectrum in a Single‐Center Cohort
Source: JIMD Rep. 2026 Oct 14;67(6):e70132. doi: 10.1002/jmd2.70132 (PMC13662738; doi:10.1002/jmd2.70132)
Supplement: Supplementary file 2 [file JMD2-67-e70132-s002.docx]

Supplementary Table 4. Spearman correlations between biochemical markers, residual enzyme activity, and metabolic decompensations in MCADD

| **Variable** | **C8-NBS (rho, p)** | **C8-CS (rho, p)** | **Enzyme activity (rho, p)** | **D/P/Y (rho, p)** |
| --- | --- | --- | --- | --- |
| C8-NBS (N=39) | — | rho=0.79; p <0.001 | rho=-0.74; p <0.001 | rho=0.25; p=0.128 |
| C6-NBS (N=28) | rho=0.96; p <0.001 | rho=0.85; p <0.001 | rho=-0.89; p <0.001 | rho=0.40; p=0.098 |
| C10-NBS (N=31) | rho=0.80; p <0.001 | rho=0.58; p=0.009 | rho=-0.71; p=0.021 | rho=0.50; p=0.018 |
| C10:1-NBS (N=24) | rho=0.91; p <0.001 | rho=0.71; p=0.009 | rho=-0.83; p=0.042 | rho=-0.04; p=0.904 |
| C8/C10-NBS (N=32) | rho=0.81; p <0.001 | rho=0.74; p <0.001 | rho=-0.78; p=0.004 | rho=0.27; p=0.214 |
| C8/C2-NBS (N=21) | rho=0.87; p=0.004 | rho=0.91; p=0.002 | rho=-0.94; p=0.005 | rho=0.11; p=0.796 |
| C6-CS (N=28) | rho=0.67; p <0.001 | rho=0.92; p <0.001 | rho=-0.82; p <0.001 | rho=0.54; p=0.003 |
| C8-CS (N=36) | rho=0.79; p <0.001 | — | rho=-0.83; p <0.001 | rho=0.40; p=0.017 |
| C10-CS (N=13) | rho=0.54; p=0.054 | rho=0.86; p <0.001 | rho=-0.72; p=0.045 | rho=0.69; p=0.009 |
| C10:1-CS (N=21) | rho=0.47; p=0.037 | rho=0.88; p <0.001 | rho=-0.82; p=0.007 | rho=0.62; p=0.003 |
| C8/C10-CS (N=12) | rho=0.53; p=0.075 | rho=0.91; p <0.001 | rho=-0.85; p=0.016 | rho=0.66; p=0.019 |
| Enzyme activity (N=20) | rho=-0.74; p <0.001 | rho=-0.83; p <0.001 | — | rho=-0.62; p=0.004 |

Data are presented as Spearman correlation coefficient (rho) and p value. N in the first column indicates the number of available values for each variable; correlations were calculated using pairwise available data. NBS, newborn screening; CS, confirmatory study; D/P/Y, metabolic decompensations per patient-year; MCADD, medium-chain acyl-CoA dehydrogenase deficiency.
