# Supplementary material for: Fatty Acid Oxidation Disorders Detected by Newborn Screening: Clinical, Biochemical and Genetic Spectrum in a Single‐Center Cohort
Source: JIMD Rep. 2026 Oct 14;67(6):e70132. doi: 10.1002/jmd2.70132 (PMC13662738; doi:10.1002/jmd2.70132)
Supplement: Supplementary file 3 [file JMD2-67-e70132-s003.docx]

Supplementary Table 2. Individual newborn screening, confirmatory biochemical, residual enzyme activity and clinical data in patients with MCADD.

| Case | NBS  C6  µmol/L  <0.10* | NBS  C8 µmol/L  <0.13* | NBS  C10 µmol/L  <0.22* | NBS  C10:1 µmol/L  <0.10* | NBS  C8/C10 µmol/L | NBS  C8/C2µmol/L | CS  C6 µmol/L  <0.13* | CS  C8 µmol/L  <0.26* | CS  C10 µmol/L  <0.35* | CS  C10:1 µmol/L  <0.22* | CS  C8/C10 µmol/L | Residual enzyme activity  (%) | Admissions per patient / year | Decompensation per patient / year |
| --- | --- | --- | --- | --- | --- | --- | --- | --- | --- | --- | --- | --- | --- | --- |
| 7 | 0.45 | 0.93 | 0.45 | 0.12 | 2.06 | 0.04 | 0.17 | 0.28 | 0.14 | 0.13 | 2.00 | 29.00 | 0.00 | 0.00 |
| 8** | 0.43 | 1.03 | 0.56 | 0.16 | 1.84 | 0.04 | 0.12 | 0.21 | 0.15 | 0.11 | 1.40 |  | 0.00 | 0.00 |
| 9 | 0.78 | 1.07 | 0.41 | 0.30 | 8.67 | 0.19 | 0.22 | 0.78 |  |  |  | 8.30 | 0.00 | 0.00 |
| 10 | 1.48 | 8.43 | 0.58 | 0.31 | 14.53 | 0.25 | 1.30 | 4.65 | 0.42 | 0.38 | 11.07 | 5.90 | 0.00 | 0.00 |
| 11 |  | 0.78 |  |  |  |  |  | 0.57 |  |  |  |  | 0.18 | 0.18 |
| 12 | 2.21 | 11.4 | 0.81 |  | 14.07 |  | 4.95 | 15.19 | 1.25 | 2.65 | 12.15 | 2.60 | 0.38 | 0.19 |
| 13 | 0.27 | 0.48 | 0.55 |  | 0.87 |  | 0.15 | 0.35 | 0.38 |  |  | 24.00 | 0.00 | 0.00 |
| 14 |  | 22.40 | 1.56 |  | 14.35 |  | 1.86 | 8.20 | 0.63 | 1.28 | 11.58 |  | 0.28 | 0.19 |
| 15** | 0.22 | 0.46 | 0.28 | 0.13 | 1.64 | 0.02 |  | 0.24 |  |  |  |  | 0.23 | 0.12 |
| 16 |  | 7.16 |  |  |  |  | 1.18 | 5.20 |  | 1.10 |  |  | 0.31 | 0.21 |
| 17 |  | 8.89 | 0.64 |  | 13.70 |  | 0.82 | 3.74 |  |  |  |  | 0.00 | 0.00 |
| 18 |  | 1.56 |  |  |  |  | 0.17 | 0.46 | 0.41 | 0.28 | 1.12 | 17.00 | 0.10 | 0.00 |
| 19 |  | 5.18 |  |  |  |  | 0.80 | 3.02 |  | 0.51 |  |  | 0.11 | 0.00 |
| 20** | 1.58 | 12.00 | 0.92 | 0.69 | 13.04 |  | 0.43 | 1.37 | 0.14 | 0.22 | 9.78 |  | 0.12 | 0.00 |
| 21 |  | 0.65 |  |  |  |  | 0.24 | 0.60 | 0.47 |  | 1.27 | 29.00 | 0.00 | 0.00 |
| 22 | 0.81 | 3.48 | 0.29 | 1.27 | 12.00 | 0.31 | 2.65 | 9.55 | 0.61 | 2.51 | 15.65 | 4.40 | 0.00 | 0.00 |
| 23 |  | 9.56 | 0.64 |  | 14.05 |  | 0.83 | 2.98 |  | 0.37 |  |  | 0.29 | 0.00 |
| 24 | 2.04 | 15.00 | 0.99 | 0.41 | 15.15 | 0.79 | 1.39 | 4.42 |  | 0.51 |  |  | 0.13 | 0.00 |
| 25 | 0.86 | 3.89 | 0.74 | 0.28 | 5.25 | 0.10 | 0.57 | 1.73 |  | 0.45 |  | 18.30 | 0.38 | 0.00 |
| 26 | 1.65 | 10.57 | 0.69 | 0.98 | 15.31 | 0.35 | 0.92 | 3.21 |  | 0.39 |  |  | 0.00 | 0.00 |
| 27 | 0.55 | 2.31 | 0.92 | 0.21 | 2.51 | 0.06 | 0.54 | 1.50 |  | 0.35 |  | 13.10 | 0.13 | 0.00 |
| 28 |  | 0.47 |  |  |  |  |  |  |  |  |  |  | 0.32 | 0.00 |
| 29 | 0.14 | 0.49 | 0.29 | 0.10 | 0.72 | 0.02 | 0.23 | 0.30 |  |  |  | 17.10 | 0.36 | 0.00 |
| 30** |  |  |  |  |  |  | 0.83 | 2.99 |  | 0.51 |  |  | 0.37 | 0.00 |
| 31 | 0.44 | 1.19 | 1.02 | 0.21 | 1.16 |  |  | 0.98 |  |  |  |  | 0.41 | 0.20 |
| 32 | 0.31 | 0.37 | 0.31 | 0.08 | 1.19 | 0.02 |  |  |  |  |  | 21.60 | 0.00 | 0.00 |
| 33 | 1.06 | 5.78 | 0.70 | 0.29 | 8.30 | 0.27 |  | 2.78 |  |  |  |  | 0.00 | 0.00 |
| 34 | 2.10 | 13.7 | 0.86 | 0.38 | 15.93 | 0.67 |  | 8.00 |  |  |  | 1.30 | 0.59 | 0.39 |
| 35 | 1.48 | 9.77 | 0.75 |  | 13.02 |  | 2.94 | 11.93 | 0.72 | 1.41 | 16.56 |  | 1.13 | 0.85 |
| 36 | 1.25 | 8.1 | 0.69 | 0.70 | 11.60 | 0.64 | 0.70 | 2.44 |  | 0.26 |  |  | 0.33 | 0.00 |
| 37 | 0.21 | 0.39 | 0.31 | 0.12 | 1.25 | 0.01 | 0.31 | 0.35 |  |  |  | 13.40 | 0.00 | 0.00 |
| 38** | 0.89 | 6.60 | 0.38 | 0.41 | 17.36 |  |  |  |  |  |  |  | 0.00 | 0.00 |
| 39 | 1.71 | 7.16 | 0.46 |  | 15.56 |  |  | 7.16 |  |  |  | 2.60 | 0.67 | 0.17 |
| 40 | 0.93 | 3.31 | 0.26 | 0.38 | 12.70 | 0.25 |  | 3.20 |  |  |  |  | 1.20 | 0.30 |
| 41 |  | 0.37 |  |  |  |  |  | 0.31 |  |  |  | 11.00 | 0.12 | 0.00 |
| 42 | 2.20 | 26.00 | 2.01 | 0.35 | 12.93 | 0.80 | 0.67 | 6.31 | 0.64 | 0.56 | 9.85 |  | 0.28 | 0.00 |
| 43 |  |  |  |  |  |  | 0.16 |  |  |  |  | 32.40 | 0.00 | 0.00 |
| 44 | 2.46 | 10.90 | 1.06 | 0.55 | 10.28 | 0.58 | 1.05 | 2.51 | 0.62 | 0.71 | 4.04 | 6.70 | 0.42 | 0.00 |
| 45 |  |  |  |  |  |  |  |  |  |  |  |  | 0.00 | 0.00 |
| 46 | 0.47 | 1.80 |  | 0.26 | 1.21 | 0.33 | 1.37 | 4.28 |  | 0.48 |  | 7.20 | 1.40 | 0.00 |
| 47 |  | 0.38 | 0.26 |  | 1.46 |  |  |  |  |  |  | 60.00 | 0.00 | 0.00 |
| 48 | 0.08 | 0.18 | 0.25 | 0.08 | 0.72 | 0.01 |  | 0.63 |  |  |  |  | 0.00 | 0.00 |

*Normal Values; ** Patients presenting with symptoms before newborn screening results were available; CS: confirmatory study; NBS: newborn screening; MCADD: medium-chain acyl-CoA dehydrogenase deficiency
