# Supplementary material for: Fatty Acid Oxidation Disorders Detected by Newborn Screening: Clinical, Biochemical and Genetic Spectrum in a Single‐Center Cohort
Source: JIMD Rep. 2026 Oct 14;67(6):e70132. doi: 10.1002/jmd2.70132 (PMC13662738; doi:10.1002/jmd2.70132)
Supplement: Supplementary file 4 [file JMD2-67-e70132-s004.docx]

Supplementary Table 1. Patients presenting with symptoms before newborn screening results were available.

| **Case** | **FAOD** | **Symptoms** | **Other perinatal circumstances** | **Onset of symptoms** | **Day of NBS results** | **Attribution to FAOD** |
| --- | --- | --- | --- | --- | --- | --- |
| 1 | LCHADD | Hypoglycemia, acidosis, hyperlactacidemia | HELLP, preterm, 33 weeks’ gestation, birth weight 1900 g, Apgar 4-7-9, required endotracheal intubation and advanced neonatal resuscitation. | Within the first hour of life | 7 | Unlikely |
| 2 | LCHADD | Acidosis, hyperlactacidemia | HELLP, twin pregnancy, preterm, 30 weeks' gestation, birth weight 1360 g, respiratory distress syndrome (RDS), neonatal sepsis. | 48 hours of life | 6 | Unlikely |
| 4 | LCHADD | Hypoglycemia, required intravenous glucose infusion | Preterm, 31 weeks’ gestation, birth weight 1590 g. | 2 hours of live | 5 | Possible |
| 5 | LCHADD | Cardiomyopathy. Severe decompensation | - | 2 hours of life | 5 | Likely |
| 6 | MADD | Hypoglycemia, tremors, hypoactivity, required intravenous glucose infusion | - | Third day of life | 7 | Likely |
| 8 | MCADD | Mild hypoglycemia | - | 3 hours of life | 17 | Unlikely |
| 15 | MCADD | Mild hypoglycemia | - | 2 hours of life | 10 | Unlikely |
| 20 | MCADD | Mild hypoglycemia | - | 6 hours of life | 3 | Unlikely |
| 30 | MCADD | Mild hypoglycemia | - | 10 hours of life | 7 | Unlikely |
| 38 | MCADD | Hypoglycemia, required intravenous glucose infusion | - | 24 hours of life | 5 | Likely |
| 55 | VLCADD | Hypoglycemia, required intravenous glucose infusion | Maternal preeclampsia, preterm, 34 weeks’ gestation, low birth weight 1624 g, Apgar 4-7-9. Neonatal resuscitation. Respiratory distress | 3 hours of life | NA | Unlikely |
| 61 | CPT1D | Poor general condition; acidosis, hyperlactacidemia | Possible neonatal sepsis | Second day of life | 6 | Possible |
| 64 | CPT2D | Hypoglycemia, hyperammonemia, seizures | - | Seventh day of life | 8 | Likely |
| 65 | CACTD | Hypoketotic hypoglycemia with lactic acidosis and hyperammonemia | Two sisters died within the first 24 hours of sudden death. | 30 hours of life | NA | Likely |

*Mild hypoglycemia: resolved with oral administration of glucose or milk; CACTD: carnitine-acylcarnitine translocase deficiency; CPT1D: carnitine palmitoyltransferase 1 deficiency; CPT2D: carnitine palmitoyltransferase 2 deficiency; LCHADD: long-chain 3-hydroxyacyl-CoA dehydrogenase deficiency; MCADD: medium-chain acyl-CoA dehydrogenase deficiency; MADD: multiple acyl-CoA dehydrogenase deficiency; VLCADD: very-long-chain acyl-CoA dehydrogenase deficiency. Likely: symptoms considered mainly attributable to the underlying FAOD; NA: not available. Possible: contribution of both FAOD and perinatal factors cannot be excluded; Unlikely: symptoms considered predominantly related to perinatal conditions.
