# Supplementary material for: Fatty Acid Oxidation Disorders Detected by Newborn Screening: Clinical, Biochemical and Genetic Spectrum in a Single‐Center Cohort
Source: JIMD Rep. 2026 Oct 14;67(6):e70132. doi: 10.1002/jmd2.70132 (PMC13662738; doi:10.1002/jmd2.70132)
Supplement: Supplementary file 5 [file JMD2-67-e70132-s005.docx]

Supplementary Table 7. Anthropometric parameters at birth and last available visit.

| FAOD | Variable | Anthropometry at birth | | Anthropometry at last visit | |
| --- | --- | --- | --- | --- | --- |
|  |  | Median (IQR) | z-score | Median (IQR) | z-score |
| LC-FAOD | Age* | Gestational Age (weeks)  38.0 (35.9 - 40.3) | | Age at last visit (years)  5.6 (2.2 - 10.7) | |
|  | Weight (kg) | 2.81 (2.26 - 3.50) | -0.50 (-1.38 - 0.16) | 23.28 (13.48 - 37.73) | 0.39 (-0.75 - 1.17) |
|  | Height (cm) | 48.0 (46.1 - 51.0) | 0.3 (-0.9 - 0.4) | 120.0 (94.0 - 154.5) | 0.6 (-0.3 - 1.3) |
|  | HC (cm) | 33.5 (31.1 - 35.0) | -0.1 (-0.9 - 0.9) | 50.7 (49.0 - 52.1) | -0.3 (-1.3 - 0.7) |
| MC-FAOD | Age* | Gestational Age (weeks)  39.0 (38.3 - 40.0) | | Age at last visit (years)  7.6 (5.1 - 9.5) | |
|  | Weight (kg) | 3.19 (2.82 - 3.44) | -0.61 (-1.27 - 0.12) | 23.80 (18.60 - 41.40) | 0.70 (-0.09 - 2.10) |
|  | Height (cm) | 49.0 (47.0 - 51.0) | -0.2 (-1.2 - 0.7) | 129.0 (110.0 - 142.0) | 1.1 (0.3 - 1.9) |
|  | HC (cm) | 33.5 (33.0 - 35.2) | -0.5 (-1.5 - 0.4) | 51.0 (50.0 - 53.0) | 0.0 (-0.8 - 0.6) |

Data are expressed as median (IQR).

* Age corresponds to gestational age at birth (weeks) and age at the last available visit (years). HC: Head circumference; FAOD: Fatty acid oxidation disorders; LC-FAOD: Long-chain fatty acid oxidation disorders; MC-FAOD: Medium-chain fatty acid oxidation disorders.
