# Supplementary material for: Fatty Acid Oxidation Disorders Detected by Newborn Screening: Clinical, Biochemical and Genetic Spectrum in a Single‐Center Cohort
Source: JIMD Rep. 2026 Oct 14;67(6):e70132. doi: 10.1002/jmd2.70132 (PMC13662738; doi:10.1002/jmd2.70132)
Supplement: Supplementary file 6 [file JMD2-67-e70132-s006.docx]

Supplementary Table 6. Newborn screening markers in patients with LCHADD, CPT1D, CPT2D and CACTD.

|  | Marker (µmol/L) | Marker (µmol/L) | Marker (µmol/L) | Marker (µmol/L) |
| --- | --- | --- | --- | --- |
| LCHAD | C16OH  NV: 0.01-0.06 | C18OH  NV: 0.00-0.03 | C16:1-OH  NV: 0.02-0.07 | C18:1-OH  NV: 0.01-0.06 |
| Case 1  Case 2  Case 3  Case 4  Case 5 | 0.94  0.40  1.53  0.49  1.05 | -  0.66  1.91  0.56  0.58 | -  -  -  -  0.41 | -  0.47  1.10  0.38  1.03 |
| CPT1 | C0  NV: 6.21-43.18 | C0/C16+C18  NV: <350 | Acylcarnitines/citruline* |  |
| Case 60  Case 61 | 169  98 | 457  616 | -  13.78 |  |
| CPT2 | C16  NV: 1.21-7 | C18  NV: 0.37-1.73 | C16:1  NV: 0.07-0.43 | C18:1  NV: 0.62-2.55 |
| Case 62  Case 63  Case 64 | 11.40  -  26.30 | 1.72  4.31  5.71 | 1.20  -  2.73 | 2.81  6.16  11.10 |
| CACT | C16  NV: 1.21-7 | C18  NV: 0.37-1.73 | C16:1  NV: 1.21-7.00 | C18:1  NV: 0.62-2.55 |
| Case 65 | 13.40 | 2.51 |  |  |

CACTD: carnitine-acylcarnitine translocase deficiency; CPT1D: carnitine palmitoyltransferase 1 deficiency; CPT2D: carnitine palmitoyltransferase 2 deficiency; LCHADD, long-chain 3-hydroxyacyl-CoA dehydrogenase deficiency; NBS: Newborn screening; NV: normal values. *C0+C2+C3+C16+C18+C18:1/citruline. NV: normal values.
