# Supplementary material for: Fatty Acid Oxidation Disorders Detected by Newborn Screening: Clinical, Biochemical and Genetic Spectrum in a Single‐Center Cohort
Source: JIMD Rep. 2026 Oct 14;67(6):e70132. doi: 10.1002/jmd2.70132 (PMC13662738; doi:10.1002/jmd2.70132)
Supplement: Supplementary file 7 [file JMD2-67-e70132-s007.docx]

Supplementary Table 5. Individual newborn screening, confirmatory biochemical and residual enzyme activity data in patients with VLCADD.

| Case | C14  NBS  µmol/L  <0.45* | C14:1  NBS  µmol/L  <0.38* | C14:2  NBS  µmol/L  <0.06* | C16  NBS  µmol/L  <7.49* | C16:1  NBS  µmol/L  <0.47* | C18  NBS  µmol/L  <1.78* | C18:1  NBS  µmol/L  <2.64* | C18:2  NBS  µmol/L  <0.46* | C14:1/ C12:1  NBS  <4.50* | C14  CS  µmol/L  <0.07 | C14:1  CS  µmol/L  <0.17* | C14:2  CS  µmol/L  <0.11* | C16  CS  µmol/L  <0.28* | C16:1  CS  µmol/L  <0.07* | C16:2  CS  µmol/L  <0.08* | C18  CS  µmol/L  <0.13* | C18:1  CS  µmol/L  <0.19* | C18:2  CS  µmol/L  <0.26* | EA  % |
| --- | --- | --- | --- | --- | --- | --- | --- | --- | --- | --- | --- | --- | --- | --- | --- | --- | --- | --- | --- |
| 49 | 1.09 | 2.78 | 0.43 | 2.60 | 0.51 | 0.96 |  | 0.33 | 14.54 | 0.34 | 1.58 | 0.56 | 0.14 | 0.15 | 0.08 | 0.07 | 0.10 | 0.06 | 2.70 |
| 50 | 0.68 | 1.25 | 0.16 |  |  |  |  |  | 11.36 | 0.19 | 0.79 | 0.23 |  |  |  |  |  |  | 3.70 |
| 51 |  | 7.55 |  |  |  |  |  |  |  | 0.42 | 1.69 | 0.65 | 0.19 | 0.14 | 0.10 | 0.06 | 0.11 | 0.08 | 1.50 |
| 52 |  | 2.65 |  |  |  |  |  |  |  |  | 1.73 |  |  |  |  |  |  |  | 9.90 |
| 53 | 2.58 | 3.30 | 0.16 | 7.49 | 1.07 | 1.98 | 3.31 | 0.18 | 4.52 | 0.28 | 0.87 | 0.25 | 0.29 | 0.13 | 0.05 | 0.10 | 0.17 | 0.08 | 8.90 |
| 54 |  | 0.78 |  |  |  |  |  |  |  |  | 0.39 |  |  |  |  |  |  |  | 11.00 |
| 55** | 1.36 | 1.75 |  | 1.89 |  | 0.62 |  |  |  | 1.10 | 5.00 | 0.76 | 0.71 | 0.46 | 0.16 | 0.62 | 1.07 | 0.28 | 3.00 |
| 56 |  |  |  |  |  |  |  |  |  |  |  |  |  |  |  |  |  |  | 18.00 |
| 57 |  | 5.88 |  |  |  |  |  |  |  | 0.45 | 2.36 | 0.40 | 0.36 | 0.40 | 0.14 | 0.15 | 0.49 | 0.21 | 5.20 |
| 58 | 0.92 | 1.28 | 0.30 | 4.26 | 0.41 | 1.17 | 1.56 | 0.30 | 6.40 | 0.34 | 1.29 | 0.50 |  |  |  |  |  |  | 28.00 |
| 59 | 0.74 | 0.95 | 0.11 | 4.85 | 0.48 | 0.92 | 1.69 | 0.13 | 1.83 |  | 0.24 |  |  |  |  |  |  |  | 30.00 |

*Normal Values; **Previous symptoms before NBS results were available. CS: confirmatory study; EA: residual enzyme activity; NBS: newborn screening; VLCADD: very long-chain acyl-CoA dehydrogenase deficiency
